# Invasive brain mapping identifies personalized therapeutic neuromodulation targets that suppress OCD network activity

**DOI:** 10.1038/s41398-025-03690-z

**Published:** 2025-10-31

**Authors:** A. Moses Lee, Audrey Kist, John Alvarez, Kristin K. Sellers, Ankit N. Khambhati, Leo P. Sugrue, Lee B. Reid, Kelly Kadlec, Sneha Ray, Joline M. Fan, Anusha B. Allawala, Caroline A. Racine, Tenzin Norbu, Dani Astudillo, Alexandra G. Tremblay-McGaw, Natalie Becker, Jessica Verhein, Ahmad Alhourani, Philip A. Starr, Edward F. Chang, Andrew D. Krystal

**Affiliations:** 1 Weill Institute for Neurosciences, University of California, San Francisco, US; 2 Department of Psychiatry and Behavioral Sciences, University of California, San Francisco, US; 3 Department of Neurological Surgery, University of California, San Francisco, US; 4 Department of Radiology and Biomedical Imaging, University of California, San Francisco, US; 5 Department of Neurology, University of California, San Francisco, US

**Keywords:** Neuroscience, Psychiatric disorders

## Abstract

Deep brain stimulation has been used to treat severe, refractory obsessive-compulsive disorder (OCD) with variable outcomes across multiple anatomical targets. To overcome these limitations, we developed an invasive brain mapping paradigm in which electrodes were implanted across the OCD cortico-striato-thalamo-cortical circuit. We then performed extensive stimulation mapping during a multi-day inpatient stay to identify personalized therapeutic targets and characterize their downstream circuit effects. We found two targets within the right ventral capsule (VC) that acutely reduced OCD symptoms. Prolonged VC stimulation suppressed high frequency activity within the structurally and functionally connected orbitofrontal and cingulate cortex, which were identified to be cortical nodes encoding the severity of OCD symptoms. These VC sites were implanted for DBS and combined stimulation of these targets led to a rapid therapeutic response. This case provides the first proof-of-concept that invasive brain mapping can be used to guide a novel personalized, multi-site neuromodulation approach to treat refractory OCD.

## Introduction

Deep brain stimulation (DBS) is a treatment for severe, refractory obsessive-compulsive disorder (OCD). Multiple DBS targets, such as the ventral anterior limb of the internal capsule, ventral striatum, bed nucleus of the stria terminalis, and subthalamic nuclei, have been used to treat OCD. The response rates of ~ 60% across sites with an average reduction in symptoms of ~ 40% [1, 2]. The lack of consistency in the response to any individual target may be due to factors such as heterogeneity in circuit abnormalities underlying the disorder, anatomical variation across individuals, and lack of target engagement. Moreover, attempts to optimize DBS for treating OCD have been limited by the lack of understanding regarding its underlying circuit-based mechanism of action. The variable response to DBS delivered to each of these sites underscores the need to develop an approaches that can identify individualized therapeutic targets to optimize outcomes for OCD [3, 4].

To address these challenges, we developed a protocol that aimed to identify personalized neuromodulation targets. Adapting methods commonly used in epilepsy to inform surgical treatment of epilepsy, our protocol involves the implantation temporary intracranial electrodes across the cortico-striato-thalamo-cortical (CSTC) circuits in a set of evidence-based candidate targets of neuromodulation for OCD. Our clinical goal was to perform extensive stimulation mapping to identify therapeutic sites that acutely reduced OCD symptoms while avoiding sites that lead to adverse effects that may limit the efficacy of chronic DBS. The invasive brain monitoring stage also provided us with a rare opportunity to identify personalized electrophysiological biomarkers of OCD symptom within the CSTC circuit and verify that acutely therapeutic stimulation modulated these biomarkers of target engagement using a multi-modal approach combining intracranial recordings and imaging. We then aimed to implant the top personalized candidate targets to implement a novel combined multi-site neuromodulation protocol that was aimed at achieving a more rapid and consistent improvement in symptoms across subjects.

Here, we present results from our first patient to undergo our protocol. The patient was a 20 s year-old woman with extreme, treatment-refractory OCD (Yale Brown Obsessive-Compulsive Scale Y-BOCS: 34). Her OCD symptoms mostly consisted of harm-based obsessions associated with checking compulsions. These symptoms were disabling, occupied most of her waking hours, caused her to leave nursing school, and prevented her from driving or leaving home. She had not responded to multiple trials of serotonin reuptake inhibitors, augmentation strategies, outpatient and intensive residential therapy, and transcranial magnetic stimulation. Given the severity and refractoriness of her symptoms, she was enrolled in this FDA-approved (IDE G240038) clinical trial (ClinicalTrials.gov NCT06347978) using invasive brain mapping to guide personalized, four-lead DBS to treat OCD.

## Materials and methods

### Ethics approval and consent to participate

The patient gave written informed consent for participant in this clinical trial of SEEG-guided personalized DBS for OCD (NCT06347978), approved by the institutional review board of UCSF and FDA. This study was conducted in accordance with the ethical standards prescribed in the 1964 Declaration of Helsinki. The participant provided signed permission to use personal health information for research and authorization for the distribution and publication of images related to the study.

### Invasive brain mapping of cstc network

**Fig. 1:**
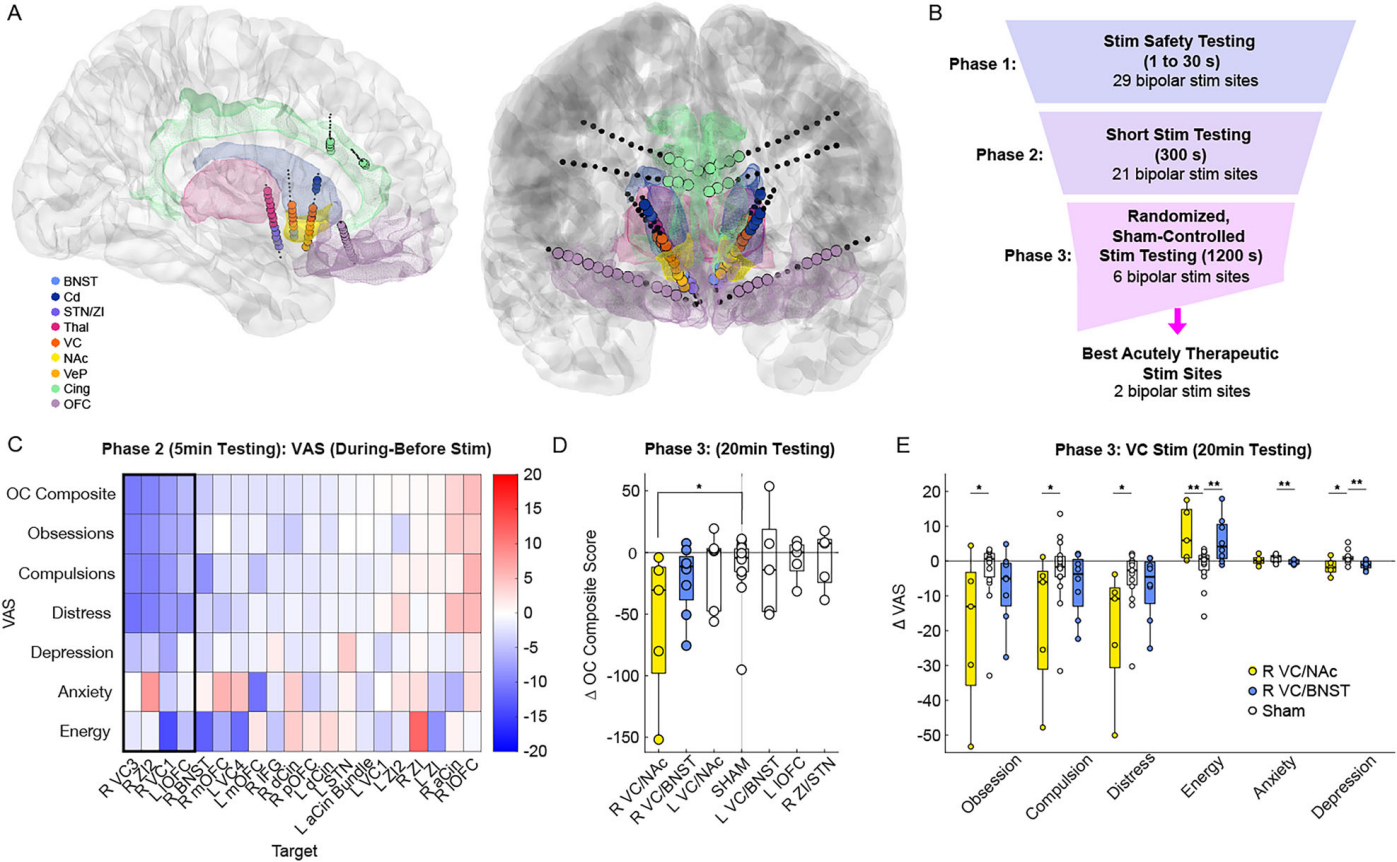
Invasive Brain Mapping to Identify Personalized Neuromodulation Targets. **A** Location of anatomically verified intracranial electrodes, **B** Stimulation testing paradigm during invasive monitoring, **C** Mean change in VAS before and during stimulation across sites during Phase 2 efficacy testing, **D**) Changes in VAS- OC composite score before and during stimulation across six top candidates sites and with sham during Phase 3, **E**) Change in VAS subscales before and during R VC/NAc, R VC/BNST, and sham stimulation during Phase 3 (* p < 0.05, ** p < 0.01, significance based upon permutation test) (left).

Twelve stereoelectroencephalography electrodes, each with sixteen contacts, were implanted bilaterally in the orbitofrontal cortex (OFC) [5], anterior cingulate cortex (ACC) [6, 7], dorsal cingulate cortex (DCC), ventral capsule/nucleus accumbens (VC/NAc) [8], ventral capsule/bed nucleus of the stria terminalis (VC/BNST) [9, 10], and anterior medial subthalamic nuclei as well the neighboring zona incerta (amSTN/ZI) [11, 12] (Fig. 1A). The post-operative CT was registered to the pre-operative MRI to verify the location of each electrode contact.

We then performed extensive stimulation mapping testing (Fig. 1B) during a 10-day inpatient monitoring stay. In Phase 1, we conducted safety testing with brief stimulation trains across anatomical regions of interest at currents ranging from 1–6 mA and durations of 1–30 s to rule out sites associated with adverse effects, such as involuntary movements, autonomic effects, excessive mood elevation concerning for mania, or spiking activity on iEEG monitoring concerning for progression to a seizure. In Phase 2, we delivered 5 min stimulation trains at contact pairs that were not associated with adverse effects to identify sites associated with improvement in self-reported OCD symptoms while again monitoring for safety. Symptoms were assessed using visual analogue scales (VAS) for obsessions, compulsions, OCD-related distress, depression, anxiety, and energy. In Phase 3, these top candidate sites were tested using a 20 min randomized, sham-controlled stimulation paradigm. Both the participant and clinicians were blinded to stimulation site, with repeated trials of stimulation. A provocation paradigm was used during testing to elicit OCD symptoms if the participant was asymptomatic.

### Identifying biomarkers of OCD symptoms and characterizing network effects of acutely therapeutic stimulation

**Fig. 2:**
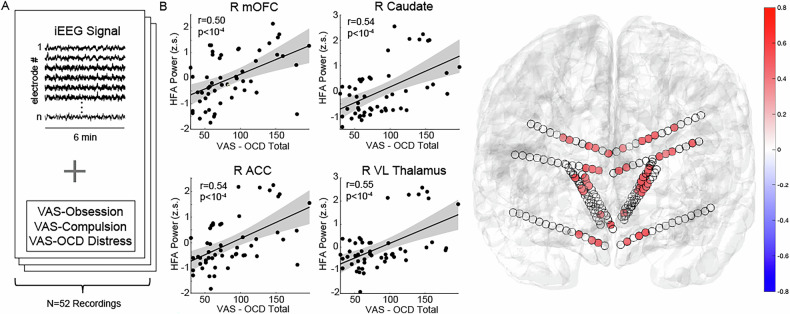
CSTC high frequency activity (HFA) correlates with OCD symptom severity. **A** Biomarker development from intracranial recordings and VAS self-report, **B** Examples of correlation between HFA and VAS-OCD total scores across representative brain regions within CSTC network (left). Shaded region represents 95% confidence interval. Summary of significant HFA vs VAS-OCD total correlations across implanted electrodes (significance defined by Bonferroni-corrected Permutation Test for 174 recording sites). Color bar represents Pearson correlation.

To characterize the relationship between CSTC activity and OCD symptoms, we collected intracranial EEG recordings during VAS assessments (Fig. 2A). An OCD composite total score representing severity of OCD symptoms was calculated as the sum of VAS scales for obsessions, compulsions, and OCD-related distress, which were all strongly correlated (Extended Data Fig. 1). We then identified electrophysiological spectral correlates of the self-report scales and composite total score.

We also sought to characterize the neurophysiological effect of stimulation at the acutely therapeutic sites on the CSTC network by identifying spectral changes before and during stimulation. We also conducted single pulse evoked potential studies to characterize the directional electrophysiological connectivity between the therapeutic stimulation sites and downstream recording sites throughout the CSTC network. We then compared this functional connectivity to the structural connectivity identified with tractography derived from diffusion-weighted MRI seeded to the region of the therapeutic contacts.

### DBS therapy

SEEG electrodes were explanted at the end of the invasive monitoring stay. Eight weeks later, the participant underwent four-lead DBS surgery with bilateral Medtronic Percept RC implantable pulse generators at sites associated with acute therapeutic improvement. Two weeks following implantation, an open-label DBS programming phase was initiated in which DBS parameters were adjusted to achieve optimal clinical improvement.

## Results

### Stimulation mapping to identify therapeutic stimulation targets

Initial stimulation safety (Phase 1) and preliminary 5 min stimulation efficacy testing (Phase 2) identified six target that were associated with self-reported improvements in VAS-OCD composite scores (Fig. 1B, C). In Phase 3, these top candidate sites were tested using a 20 min randomized, double-blinded, sham-controlled stimulation across repeated trials of stimulation (Fig. 1B, D left). During this phase, two targets within the right ventral capsule (VC), above the neighboring the NAc and BNST and adjacent to the globus pallidus externa, were associated with the most improvement in OCD symptoms (Fig. 1D, E) (Extended Data Fig. 2). VAS changes in energy, anxiety, and depression were also observed, though these were small relative to the improvement in OCD symptoms with sham-controlled stimulation. The participant primarily noticed a decrease in the frequency and distress related to her obsession with stimulation at both of these targets. In general, the right VC/NAc and VC/BNST SEEG leads were positioned slightly lateral relative to their left-sided counterparts, perhaps explaining the difference in acute therapeutic efficacy.

### Electrophysiological biomarkers of OCD symptom state

We then wanted to identify electrophysiological biomarkers of OCD severity, focusing in particular on high frequency activity (HFA: 30–95 Hz), which is a measure of local neuronal population activity (Fig. 2A). We found that HFA within the orbitofrontal cortex, anterior/dorsal cingulate, caudate, and ventral thalamus correlated significantly with the OCD total score (Fig. 2B). We also found that beta and mid-frequencies throughout the CSTC were correlated with the severity of OCD symptom whereas low-frequency (delta) power at some CSTC sites was anti-correlated with OCD symptoms (Extended Data Fig. 3) consistent with prior studies [13]. Together, these data are consistent with the hypothesis that higher activity within a distributed CSTC circuit is associated with greater severity in OCD symptoms.

### Therapeutic stimulation modulates cortical nodes of OCD network

**Fig. 3:**
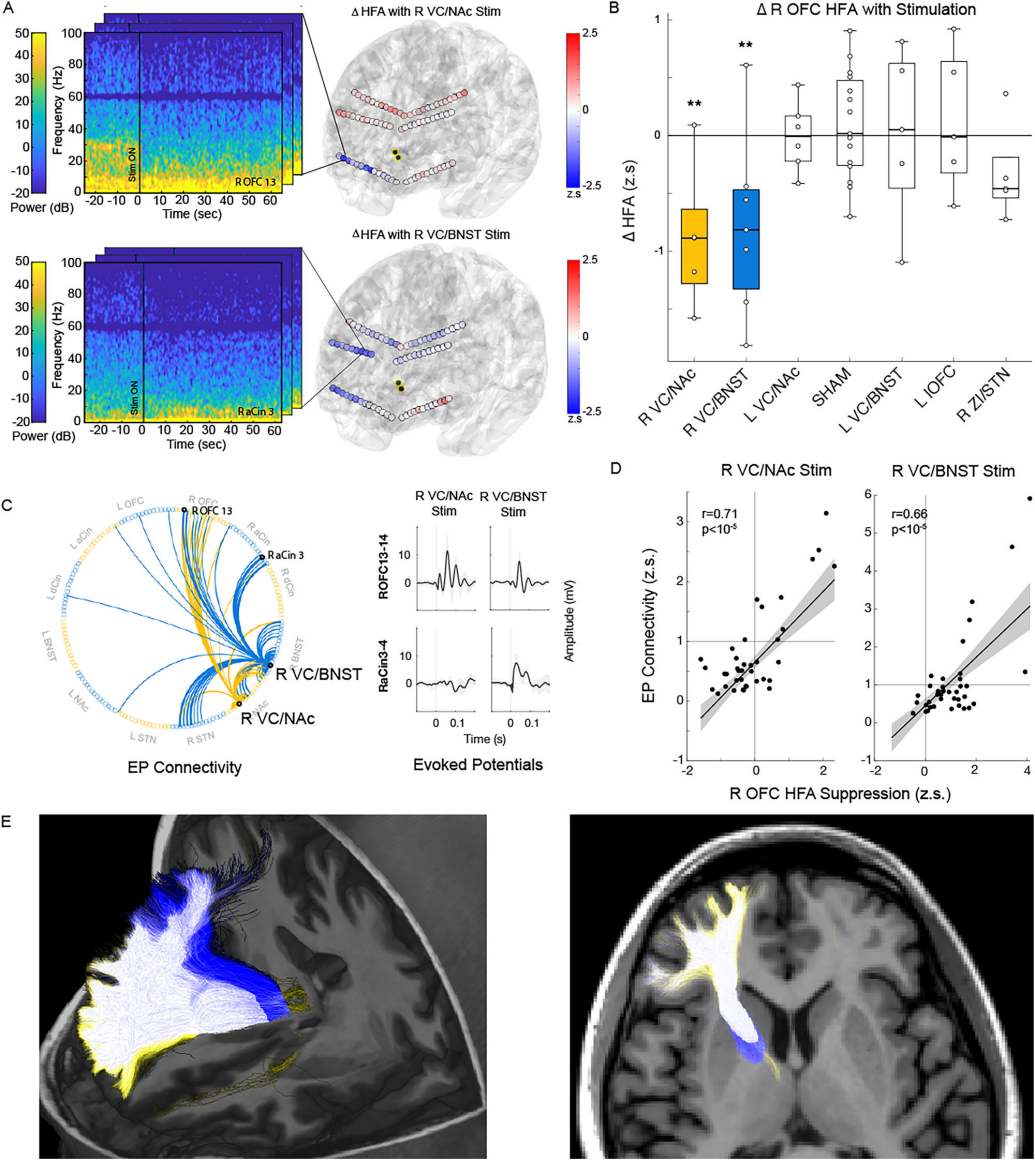
Acute therapeutic stimulation in ventral capsule suppresses orbitofrontal HFA. **A** Example spectrogram of R lateral orbitofrontal spectral changes with R VC/NAc stimulation (top left). Example spectrogram of R anterior cingulate spectral changes with R VC/BNST stimulation (bottom left). Summary of change in cortical HFA (z-scored to baseline) with stimulation during 20 min recordings across cortical recording sites (right). Contacts highlighted in yellow represent stimulation contacts. **B** Trial-by-trial change in average HFA across right orbitofrontal recording sites with stimulation at candidate targets (** p < 0.01, permutation test comparing active stimulation to sham, 20 min recordings). **C** Directed graph of single-pulse stimulation N1 (10–50 ms post-stimulation) EP responses (greater than 1 standard deviation) from R VC/NAc (yellow) and R VC/BNST (blue) contacts with examples of EP waveforms. **D** Correlation of N1 EP magnitude and HFA suppression across cortical electrodes. Line represents linear fit and shaded grey region is 95% confidence interval. **E** Tractography derived from the R VC/NAc (yellow), R VC/BNST (blue), or both contacts (white).

We next wanted to characterize the neurophysiological effect of stimulation at the acutely therapeutic R VC/NAc and R VC/BNST sites on this CSTC HFA biomarker of OCD severity. Stimulation at each target was associated with a significant suppression of OFC HFA and a shift in power to lower frequencies (Fig. 3A, B, Extended Data Fig. 4). In addition, R VC/BNST also led to suppression in the anterior cingulate (Fig. 3A). The suppression of OFC activity was specific to stimulation at the R VC/NAc and R VC/BNST sites and not observed with stimulation at other candidate targets (Fig. 3B).

We then sought to understand the electrophysiological connectivity between the right candidate VC sites and OFC, ACC, and other components of the CSTC network as another measure of target engagement. We measured the N1 component of the evoked potential (EP) response across recording sites in response to single pulse stimulation of the R VC/NAc and R VC/BNST targets (Fig. 3C). Both R VC/NAc and R VC/BNST stimulation elicited strong N1 EP responses within the OFC. In addition, the R VC/BNST also had strong EP connectivity to the ACC. These results suggest that both therapeutic VC stimulation sites provide electrophysiological input to the OFC (Fig. 3C). Notably, the strength of the EP response correlated with the magnitude of suppression of high frequency activity with stimulation across cortical sites (Fig. 3D).

Lastly, we used tractography from diffusion-weighted MRI to determine structural connectivity from the therapeutic VC sites. Both sites were located within a component of the anterior thalamic radiation (ATR), which contains tracts connecting anterior thalamus with the OFC (Fig. 3E, Extended Data Fig. 5). The VC/BNST stimulation site also had dense connectivity to the ACC similar to the spatial pattern of EP and stimulation-induced suppression.

### Multi-site DBS to treat OCD

**Fig. 4:**
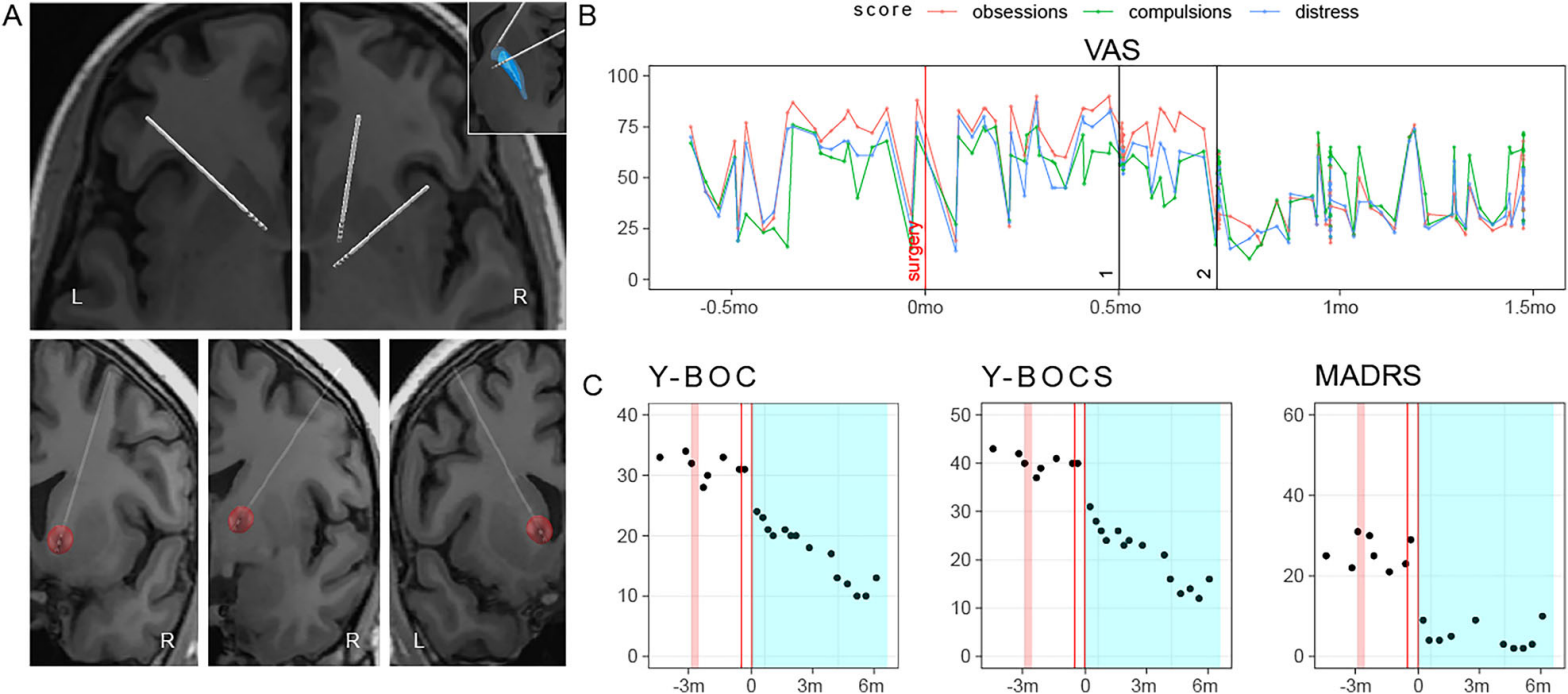
Reconstruction of Implanted DBS Leads and Clinical Response to DBS. **A** Reconstruction of DBS leads. Horizontal section demonstrating placement of left VC/NAc lead (top left panel). Horizontal section demonstrating placement of right VC/NAc as well as right VC/BNST leads (top right panel). Inset displaying right lead position relative to globus pallidus externa (top right inset). Coronal sections demonstrating placement of right VC/NAc (bottom right panel), right VC/BNST leads (bottom middle panel), and left VC/NAc lead (bottom left panel). **B** Changes in self-reported visual analog scales (VAS) for OCD symptoms over time relative to DBS implant. Black lines show the dates of changes in DBS contact configurations. For session 1, stimulated targets were R VC/NAc and L VC/NAc only at the C + 1- contacts. Starting session 2, R VC/BNST stimulation at the C + 1- contacts was added to R VC/NAc and L VC/NAc. **C** Changes in clinical Y-BOCS, Y-BOCS2, and MADRS scores over time relative to inpatient brain mapping, DBS implantation, and initial DBS programming.

In second stage of our protocol, 4 DBS leads are implanted for the purpose of therapeutically stimulating or performing longitudinal recordings to identify a biomarker of OCD symptoms. The R VC/NAc and R VC/BNST sites adjacent to the globus pallidus externa were implanted with chronic DBS leads as the most effective targets during the invasive brain mapping phase (4 A). The estimated volume of activated tissue was identified for each of the targets, and tractography was performed confirming that the DBS sites had white matter connectivity as the R VC/NAc and R VC/BNST targets identified during the invasive monitoring phase. Leads were also placed within the L VC/NAc (Fig. 4A), which had shown a modest but less reproducible improvement in OCD symptoms compared to the R VC sites (Fig. 1D). The left ACC was implanted for long-term chronic sensing using the Percept device. Initial DBS programming took place two weeks after DBS surgery. DBS was first delivered bilaterally to VC/NAc, similar to the conventional DBS configuration for OCD, resulting in a modest improvement in OCD and mood symptoms. The following week, the personalized R VC/BNST DBS was added with no other changes being made to bilateral VC/NAc sites, leading to a larger improvement in OCD as measured by VAS with combined stimulation (Fig. 4B). Overall, VAS improvements were highly correlated with assessments of weekly Y-BOCS improvements (Extended Data Fig. 6). After an initial drop in symptom severity, an additional gradual improvement in OCD symptoms was observed over time, reaching a plateau after 4–6 months. In contrast, depression improvements occurred more rapidly (Fig. 4B). Six months from initial programming, the participant experienced a 62% improvement in her YBOCS score (Y-BOCS-1 13) compared to baseline (Y-BOCS-1 34) (Fig. 4C). In the third phase of the trial, a brief washout was conducted prior to the blinded randomization to active or sham conditions. During the washout, OCD symptoms rapidly returned to within 6% of the baseline level of symptoms (YBOCS-washout: 32).

## Discussion

Here, we describe an invasive brain mapping paradigm involving extensive stimulation testing to identify acutely therapeutic stimulation sites to guide multi-site DBS to treat severe, refractory OCD. The inpatient invasive monitoring stay also allowed for a rare opportunity to conduct intracranial recordings to identify electrophysiological biomarkers of OCD symptom severity and therapeutic response to stimulation. In our first case, we identified two targets within the right VC nearby the NAc and BNST were identified that rapidly relieved the participant’s self-report OCD symptoms. We also found that HFA distributed throughout the CSTC network was correlated with OCD symptoms. Stimulation at our two acutely therapeutic VC sites suppressed HFA within the OFC. Evoked potential mapping for our therapeutic sites demonstrated dense electrophysiological connectivity between these components of the VC and the OFC, and structural connectivity derived from diffusion-weighted imaging verified that the therapeutic sites are located within the ATR connecting anterior thalamus to OFC. Subsequently, the best candidate stimulation sites identified during the invasive brain mapping was used to inform a multi-site DBS approach that lead to rapid improvement in the participant’s OCD symptoms. The magnitude of the symptom reduction of 62% based upon the YBOCs at 6 months is larger than the average reported in meta-analysis of ~ 35% [14].

These results of this case are consistent with models hypothesizing that elevated CSTC network activity mediates OCD symptoms [15–17]. This finding is consistent with prior studies that have noted a similar pattern of enhanced striatal HFA and suppressed low frequency activity with elevated OCD symptoms [13, 18]. In this model, therapeutic VC stimulation disrupts basal thalamocortical input, reducing activity in the orbitofrontal and cingulate cortices, leading to reductions in OCD symptoms [19, 20]. Prior studies have demonstrated that stimulation near the ATR projecting to OFC is associated with better DBS outcomes in OCD [21–25]. This model is consistent with theories that VC DBS acts as a reversible informational lesion [26] and the existing clinical evidence that capsulotomy can effectively treat refractory OCD [27, 28].

This case provides the first proof-of-concept that acute stimulation at specific targets can rapidly and reproducibly reduce OCD symptoms during an invasive monitoring stay and be used to guide multi-site DBS. Our observation of OCD symptom reduction within a short timeframe is notable, as prior reports suggest that the ‘wash-in’ time for OCD benefit from DBS typically takes days to weeks. In fact, most intra-operative testing and VC DBS programming assess changes in ‘mood’, ’energy’, and ‘anxiety’ since changes in core OCD symptoms are not believed to be discernable during such short time frames [29]. Our ability to detect rapid changes in OCD symptoms may be due to several factors. First, the 10-day invasive monitoring period allowed us to test a wide range of stimulation contacts and configurations for longer durations than a standard outpatient visit. This provided a unique opportunity to identify stimulation settings that yielded robust immediate effects on OCD symptoms. Second, we planned symptom provocations during this monitoring period to better control OCD symptoms, disentangling therapeutic benefits specific to OCD from other confounds like mood improvements. Lastly, the randomized sham-controlled testing allowed us to identify therapeutic effects specifically due to the stimulation, as opposed to other confounds such as non-specific placebo effects or natural habituation of symptoms. The ability to extensively test and assess a wide range of stimulation settings to identify optimal parameters during an invasive brain mapping stage to guide the development on chronic DBS opens up the possibility of personalized neuromodulatory therapies.

Further, our invasive brain mapping approach allowed us to identify biomarker of OCD symptom severity and verify that our personalized neuromodulation targets are engaging these circuits. While several studies have implicated CSTC pathophysiology in OCD, this is the first study to directly demonstrate that temporal shifts in the electrophysiological activity across the CSTC network strongly correlate with the symptom severity over time within an individual. Moreover, both VC targets demonstrated strong structural and directed functional connectivity to OFC, and stimulation of these sites induced a strong suppression of orbitofrontal activity were associated with acute symptomatic improvement, suggesting a critical role of OFC in mediating symptoms within the CSTC circuit. It is possible that our novel stimulation paradigm targeting both VC/NAc and VC/BNST within thalamocortical tracts was able to more effectively suppress orbitofrontal activity than could be achieved with stimulating either site alone. This could explain why the combined multi-site stimulation strategy yielded a greater acute improvement in symptoms than standard bilateral VC/NAc DBS in this subject. In future participants, we will determine if EP connectivity to OFC and acute suppression of lateral OFC HFA with VC stimulation can serve as a physiomarker of OCD circuit engagement and a predictor of therapeutic response. If so, such biomarkers of target engagement, in combination with pre-operative diffusion tractography, could be used intra-operatively to optimize DBS targeting for OCD.

This study has some limitations. First, the stimulation mapping protocol inherently favors stimulation targets that can produce a rapid effect. Given that the inpatient monitoring stay only lasts 10 days and the large number of sites that were tested, we could only trial stimulation for a limited duration of time at each target. It is possible that candidate sites with slower onsets of response, which could still be therapeutically beneficial with chronic stimulation, may be missed in our stimulation testing paradigm. Second, it is unclear whether the biomarkers of OCD symptoms and therapeutic response that we observed during the invasive brain monitoring will generalize to the naturalistic context of the home environment. With DBS devices capable of performing longitudinal intracranial recordings and delivering stimulation, we can determine if biomarkers and stimulation findings from inpatient monitoring will generalize to real-world, long-term treatment [30–32]. We aim to address these questions in subsequent phases of this study. Lastly, one could argue that comparable outcomes could have been achieved without the need for invasive brain mapping in this participant’s case because the best candidate sites that we identified in this individual are traditional VC targets for OCD. However, it is notable that the optimal targets identified during the invasive mapping phase were closer to the globus pallidus externa, which is not a traditional OCD DBS target, as opposed to the BNST or NAc proper. Without the invasive brain mapping to guide placement of the DBS implant, it is not clear if this location within the VC would have been implanted based upon a more conventional lead placement in which the tip of the electrode is placed within the BNST or NAc. More importantly, approximately forty percent of patients do not respond to DBS targeted to these regions, and it is possible that an alternative target may be more beneficial in these non-responders. It is interesting to note that stimulation of another evidence-based target the amSTN [33] did not have any acute therapeutic response within this individual and was associated with significant side effects even at relatively low currents, which would have limited the efficacy of DBS therapy.

For this reason, it remains to be seen whether the personalized stimulation sites and biomarkers identified in this subject will generalize to other participants. If the personalized targets generalize across participants, this could indicate that there may be a common circuit dysfunction underlying OCD that can be consistently targeted to achieve therapeutic responses. This would mean that a limited number of DBS targets could treat OCD without the need for additional personalization. Alternatively, we may find that the optimal stimulation sites and OCD biomarker network are distinct across individuals. This could indicate the need for a precision medicine approach, relying on mapping for personalization of therapy to optimize outcomes. Additional cases should allow us to distinguish between these possibilities.

## Supplementary information

Supplemental Materials

## Data Availability

The data used to produce the results and figures in this paper are available upon request.
