# Supplementary material for: Invasive brain mapping identifies personalized therapeutic neuromodulation targets that suppress OCD network activity
Source: Transl Psychiatry. 2025 Oct 31;15:448. doi: 10.1038/s41398-025-03690-z (PMC12578982; doi:10.1038/s41398-025-03690-z)
Supplement: Supplementary file 1 — Supplemental Materials [file 41398_2025_3690_MOESM1_ESM.docx]

**Invasive Brain Mapping Identifies Personalized Therapeutic Neuromodulation Targets that Suppress OCD Network Activity**

## Supplementary Materials

## Methods:

## Surgical procedure

The patient gave written informed consent for participation in a clinical trial of Personalized DBS for OCD Guided by Stereoencephalography Mapping ([NCT06347978](https://clinicaltrials.gov/ct2/show/NCT06347978)), approved by the UCSF institutional review board and Food and Drug Administration (FDA). The patient was surgically implanted with twelve stereoelectroencephalography (SEEG) electrodes (PMT Corporation) within the most promising sites bilaterally for modulating OCD based on published literature: VC/NAc [2], VC/BNST [9, 10], and amSTN/ZI [11, 33]. Additionally, we targeted anterior and posterior dorsal sites in the ACC as well as the OFC as these regions are the targets of TMS for OCD [5, 6] and cingulotomies [34]. SEEG leads with 2.5mm center-to-center spacing were used in targeting the subcortical VC/NAc, VC/BNST, and amSTN/ZI, and leads with 3.5mm spacing were used in ACC and OFC. Surgical targeting was planned in Brainlab iPlan Cranial Software using a combination of anatomic and diffusion tractography based targeting. Computed tomography (CT) was used intra- and post-operatively to confirm electrode placement. No complications of surgery occurred. Post-operative CT was registered to pre-operative MRI to determine the anatomic location of each electrode contact. All electrode locations were analyzed by a neuroradiologist (LS), who confirmed that a subset of the 16 contacts on each lead was in the anatomic area of interest. Exploratory intracranial stimulation and recording took place over a 10-day period. At the end of the 10-day hospitalization, the electrodes were explanted.

## Stimulation testing

Briefly, we conducted safety stimulation testing of a preselected set of 49 stimulation targets through a bipolar stimulation survey, using charge-balanced biphasic stimulation with a frequency of 100 Hz and pulse width of 100 μs. Stimulation current amplitudes and durations were progressively increased from 1–6 mA for 1,3, and 30 seconds. After an initial broad survey, we selected a reduced set of 21 sites for 5-minute preliminary stimulation efficacy testing again at progressively higher currents from 2-6 mA. The six stimulation configurations associated with the greatest acute improvement in OCD symptoms were then tested during longer stimulation periods (20 min) with randomized blinded, sham-controlled stimulation in which the assessing clinicians and participant were unaware of the stimulation parameters being tested.

**Clinical measures**

We measured symptoms intensity by continuously collecting visual analog scales (VAS) using REDCap electronic data capture tools hosted at UCSF. We collected VAS-scales for: Obsessions, Compulsions, OCD-related Distress, Anxiety, Depression, and Energy. They were measured on a continuous scale from 0 to 100. We found that OCD sub-scores were highly correlated (Extended Data Fig 1). As a consequence, we used a composite total score summing all the scores (obsessions, compulsions, OC-distress) to reduce the dimensionality of the analysis, when necessary.

To select the most promising targets during Phase 2, we collected the mean VAS value during the 5 minutes preceding the start of the stimulation (baseline score) and the mean VAS during the 5 minutes of stimulation. We ranked targets by order of best change in a single recording, i.e. biggest decrease in OC composite score (Fig. 1C). To assess the effect of stimulation on the scores, we collected the mean VAS value during the 5 minutes preceding the start of the stimulation and the mean VAS during the 20 minutes of stimulation, including sham stimulation sessions. We extracted the change in VAS for all trials (N= 5 to 9 trials depending on stimulation target, 15 sham trials). We compared the change due to the stimulation of each target to the change due to sham stimulation using permutation tests with 10,000 permutations (Fig. 2D, E).

## iEEG recording

Intracranial electroencephalogram (iEEG) recordings were collected from implanted SEEG electrodes. Stimulation through these electrodes either as single pulses or continuous stimulation was used to assess connectivity and assess for clinical and network-wide effects. iEEG recordings and evoked potentials mapping were acquired using a 256-channel Nihon Kohden clinical system and secondary data stream at a sampling rate of 10 kHz.

**Biomarker Analysis**

iEEG recordings were divided into biomarker sessions, each consisting of 6-minute segments aligned with the time periods immediately preceding each symptom self-report. iEEG timeseries was first re-referenced to a bipolar configuration. Data was then downsampled to a rate of 1024 Hz before undergoing high pass filter above 0.5Hz. A notch filter was then applied to remove line noise artifact at 60Hz and harmonics (120Hz, 180Hz, 240Hz).

Preprocessed data was then analyzed in the time-frequency domain using a Morlet transform. A Morlet wavelet with 6 cycles was applied, using 50 logarithmically spaced frequency filters between 1 and 250 Hz. Time-varying spectral power was then calculated across each of the 50 center frequencies. An automatic artifact rejection procedure was then employed by identifying time segments with broadband power exceeding five standard deviations from the median of the robust z-score distribution. These timepoints were excluded from further analysis. For the remaining clean time segments within each biomarker session, median spectral power was calculated for each center frequency. Frequency band-specific power was then calculated by averaging the median spectral power for all center frequencies falling within the bounds of each canonical frequency band (Delta: 1-4Hz, Theta: 4-8Hz, Alpha: 8-13Hz, Beta: 13-30Hz, HFA: 30-95Hz).

Now having a representation of median band-specific power within each frequency band for each biomarker session (6-minutes directly preceding each self-reported VAS rating), we explored the relationship between each channel’s power across time with fluctuations in the patient’s self-reported OCD symptoms. We did this by calculating the Pearson correlation between each channel’s frequency-specific powers and VAS-Composite score (calculated by summing three VAS-OCD sub-scores). We employed a permutation test to assess the statistical significance of these correlations, permuting the temporal order of VAS-OCD Composite scores 10,000 times. After generating a null distribution of correlation coefficients, the p-value for the observed correlation coefficient was calculated.

**Stimulation Effects Analysis**

iEEG recordings were segmented into blocks of time spanning 5 minutes before to 20 minutes after the onset of stimulation and downsampled to 512Hz. Signals were visually inspected to exclude trials exhibiting poor signal. A notch filter was then applied to remove line noise artifact at 60Hz and harmonics (120Hz, 180Hz, 240Hz) and re-referenced to a bipolar montage.

To assess the distributed electrophysiological effect of the stimulation in cortical areas, we first bandpass-filtered the time course of each channel’s signal into an HFA band (30-95Hz), which is known to reflect local activity. The 95Hz upper limit was used to avoid the artifactual effects of the 100Hz stimulation frequency. We calculated for each stimulation segment (time resolution = 5 s, sampling frequency = 512 Hz, frequency resolution = 0.2 Hz; figure 2C, left) (see extended Fig 3). Power spectra were then z-scored to the 30 second baseline prior to stimulation and averaged across the HFA band. The change in HFA before and during stimulation was calculated from the difference in the average power comparing the 30 seconds prior to and 20 minutes after the onset of stimulation across contacts for the R OFC lead for each stimulation trial. For each stimulation target, elicited changes were then compared to changes from sham trials using a permutation test (n = 10000, figure 2D).

## Diffusion Tractography

We acquired presurgical magnetic resonance imaging on a GE 3T Signa Premier with a 32 channel head coil, including a T1-weighted 3D BRAVO (1mm isotropic; repetition time 6.22ms, echo time 2.468ms, inversion time 600ms) and diffusion weighted dual spin echo HARDI sequence (4 b=0 and 83 b=2000s^2^/mm diffusion encoding directions split across a blip-up and blip-down acquisition; FOV 220mm, acquisition matrix 110x110, slice thickness 2mm). Diffusion weighted image noise and Gibbs ringing artefacts were reduced using MRTrix3, followed by correction for spatial distortions and head-motion via TOPUP and Eddy. MRtrix Single-shell Multi-Tissue separated the white matter signal from that of the grey matter and cerebrospinal fluid, identifying single-orientation voxels using the Dhollander method and modelling fiber orientation distributions using spherical harmonics. The T1 weighted image was N4 bias corrected, skull stripped using HD-BET, and its rigid transform calculated to both the post-operative CT and the first pre-operative distortion-corrected b=0 diffusion image. Coordinates for leads in the VC/BNST activating the lateral OFC were identified on a post-operative CT image and transformed into 2mm-radii spheres of presumed tissue activation in diffusion space, via the aforementioned rigid registrations. Tractography using iFOD2 generated 50,000 streamlines unidirectionally seeded from these regions of presumed tissue activation, that passed through a coronal plane in the frontal lobe ~7mm anterior to these spheres and terminated according to default criteria. For confirmation, we also performed tractography in the reverse direction, from the lateral OFC to these lead spheres, expecting that, should this connection be implausible, processing would take an extremely long time to complete and/or result in a high proportion of aberrant streamlines.

## Evoked Potentials Connectivity

Briefly, single-pulse evoked potential (EP) is a functional connectivity measure that describes the directional influence of one brain region on another. Intracranial EEG and intracortical stimulation offer a rare opportunity to probe directional connectivity through the delivery of a single brief stimulation pulse and examination of the evoked potentials at other sites. Evoked potential mapping was performed on day 1. We delivered bipolar single pulse stimulation at 3mA and 1 Hz for 20 s to adjacent contact pairs in each brain region.

We quantified the evoked potential responses to single pulse stimulation at all other contacts by *z*-scoring voltage against 50 ms of pre-stimulus baseline per trial, calculating the mean z-scored evoked potential across 20 trials by measuring the root mean square power of the averaged evoked potential within N1 (10–50 ms) time window. We then applied a threshold on evoked potentials with a magnitude greater than one to generate a directed network graph.

## Provocation Paradigm

Prior to and during the invasive brain monitoring stay, the participant worked with a psychologist and the team psychiatrist to develop a hierarchy of provocative exposures based upon the participant’s harm-based obsessions to elicit OCD symptoms. If the participant’s OCD symptoms declined during stimulation testing, a provocation was performed to increase symptoms to a clinical range of severity that allowed stimulation settings to be trialed so a therapeutic benefit could be observed.

**DBS Surgery and Programming**

Medtronic Sensight DBS leads (B33015) were implanted in the right VC/NAc, right VC/BNST, left VC/NAc, and left ACC and connected to bilateral Percept RC IPGs. Right leads were connected to the right IPG, and the left leads were connected to the IPG. Two weeks later, initial DBS programming was performed. Stimulation was delivered continuously at 130Hz between 7-9mA.


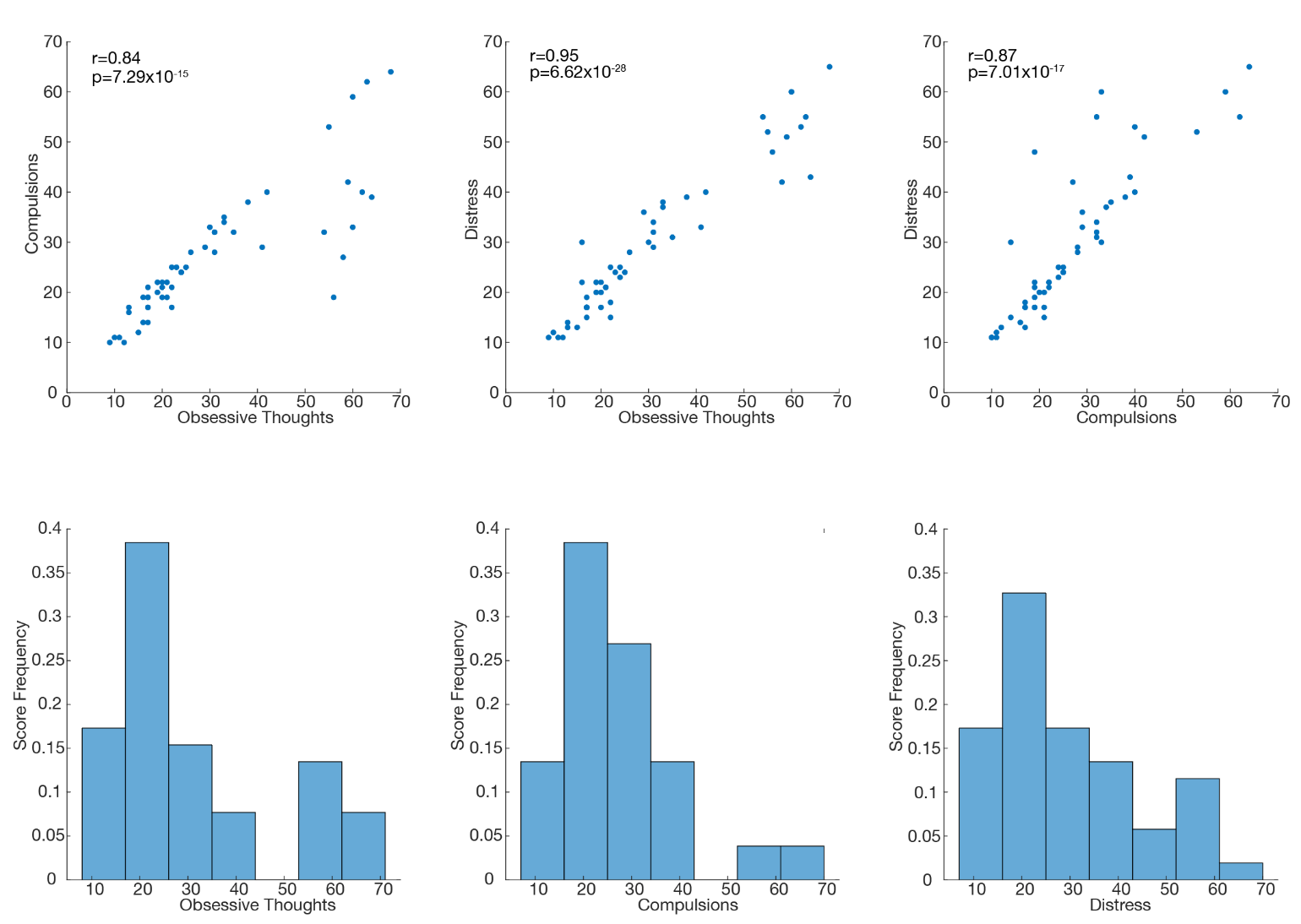


## Extended Data Fig 1: VAS OCD Subscales

Correlation between OCD subscale scores (top). Histograms of OCD subscale scores (bottom)


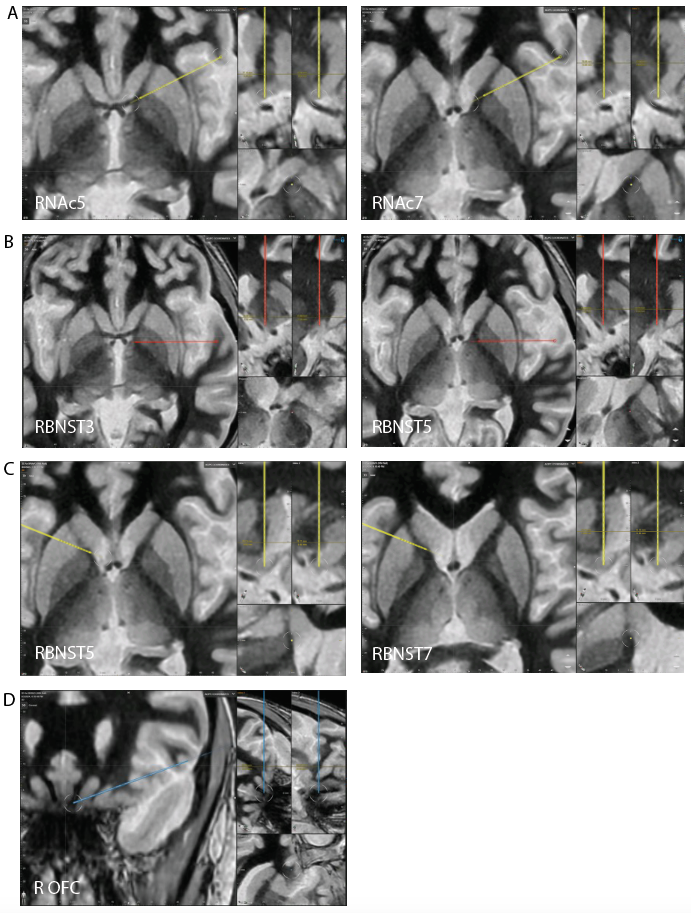


## Extended Data Fig 2: Locations of Therapeutic Contacts and R OFC SEEG Lead

A) Location of therapeutic R VC/NAc contacts (coronal and inline views). B) Location of therapeutic R VC/BNST contacts (coronal and inline views). C) Location of L VC/NAc contacts (coronal and inline views). D) Location of R OFC SEEG lead.


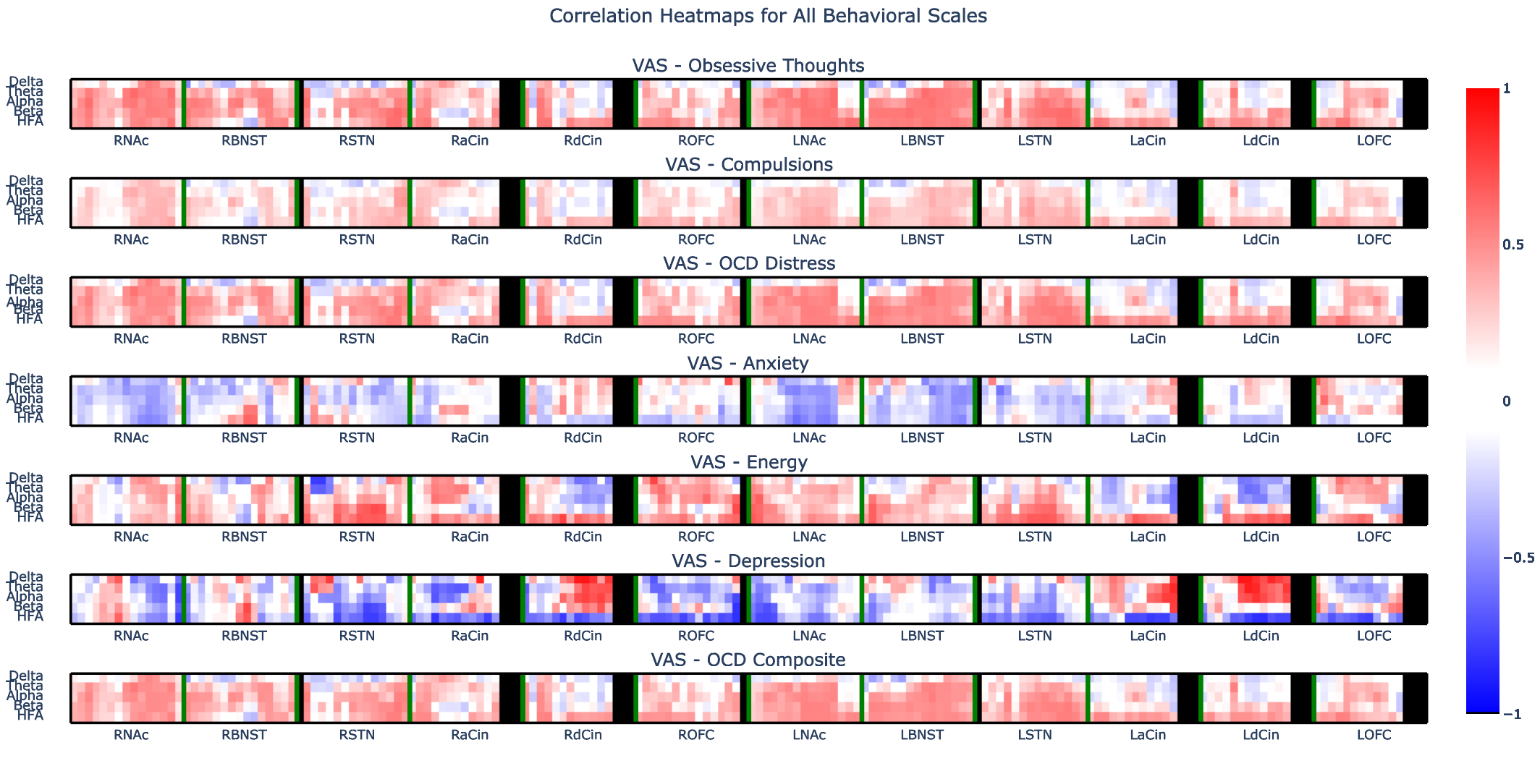


## Extended Data Fig 3: Correlation between VAS scores and power across recording sites

Heatmap of correlation between visual analogue scales for obsessive thoughts, compulsions, OCD distress, anxiety, energy, depression, and the OCD total composite score and power in the delta, theta, alpha, beta, and HFA (30-95-Hz) bands. Red indicates positive correlation. Blue indicates anti-correlation.


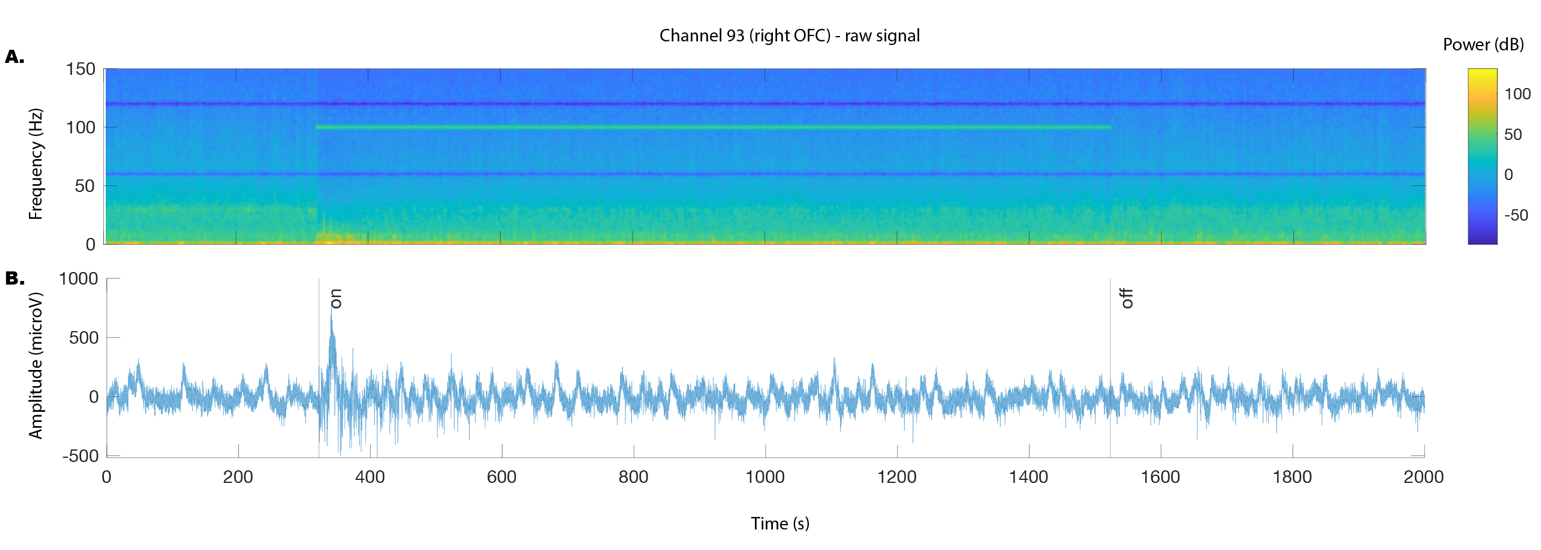


## Extended Data Fig 4: Example recording of an RVC/NAc stimulation trial. Example A) spectrogram and B) LFP recording for right lateral OFC recording (same electrode as in Fig 2C) during 100Hz stimulation. Preprocessing for LFP recordings includes notch filter at 60 and 120 Hz, removal of high amplitude artifacts. Black lines indicate stimulation start and stop.

## **
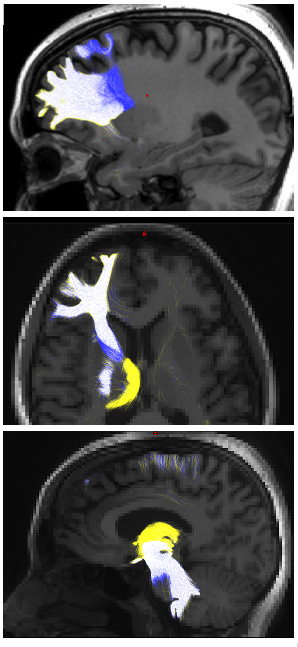
**

## Extended Data Fig 5: Tracts seeded to acutely therapeutic VC/NAc (yellow) and VC/BNST (blue) sites. White represents overlapping tracts.

## **
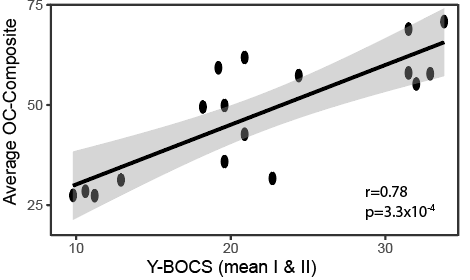
**

## Extended Data Fig 6: Validation of VAS with YBOCS. Average VAS-OCD composite scale was correlated with weekly Y-BOCS assessments.


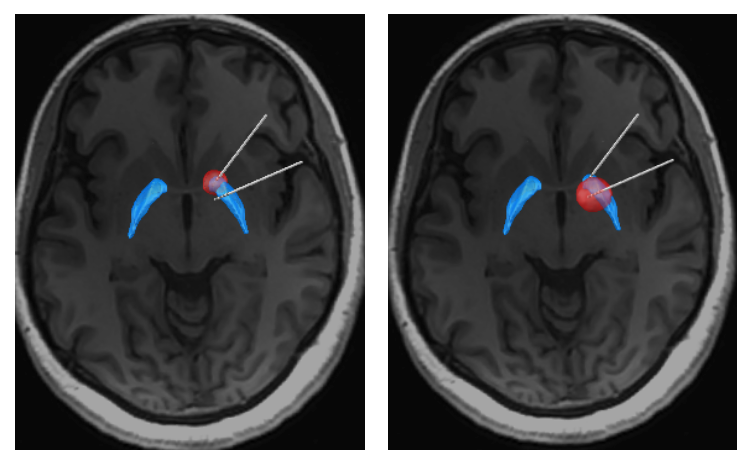
**
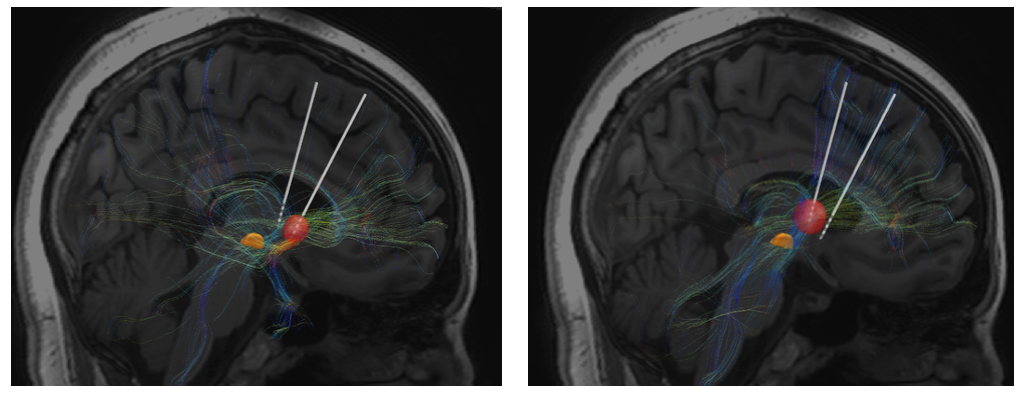
**

## Extended Data Fig 7: Tractography from DBS Stimulation sites. Volume of activated tissue (VAT) for R VC/NAc (top left) and R VC/BNST (top right) stimulation sites relative to GPe (blue). Structural connectivity from VATS seeding the R VC/NAc (bottom left) and R VC/BNST (bottom right) sites relative to STN (orange).
